# Steamed *Panax notoginseng* Saponins Ameliorate Cyclophosphamide-Induced Anemia by Attenuating Gut-Liver Injury and Activating the cAMP/PI3K/AKT Signaling Pathway

**DOI:** 10.3390/nu17213335

**Published:** 2025-10-23

**Authors:** Cuiping Xu, Hao Cui, Qionglian Fang, Pengfei Tu, Xiuming Cui

**Affiliations:** 1 Faculty of Life Science and Technology, Kunming University of Science and Technology, Kunming 650500, China; luckycuipi@163.com (C.X.);; 2 Southwest United Graduate School, Kunming 650500, China; 3 Yunnan Key Laboratory of *Panax notoginseng*, Kunming 650500, China; 4 State Key Laboratory of Natural and Biomimetic Drugs, School of Pharmaceutical Sciences, Peking University, Beijing 100191, China; 5 Laboratory of Sustainable Utilization of *Panax notoginseng* Resources, State Administration of Traditional Chinese Medicine, Kunming 650500, China

**Keywords:** SPNS, cyclophosphamide, anemia, liver-intestinal axis, metabolomics, proteomics, metagenomics

## Abstract

**Background:** Steamed *Panax notoginseng* saponins (SPNSs) can alleviate cyclophosphamide-induced anemia. However, the hepatointestinal effects of SPNSs and their role in ameliorating cyclophosphamide-induced anemia remain unexplored. **Objective:** To elucidate the hepatointestinal effects of SPNSs and their role in ameliorating cyclophosphamide-induced anemia. **Methods:** Blood samples were collected and analyzed on days 7 and 14. Liver tissues and small intestinal villi structures were observed via HE staining. Liver and colon content metabolites were detected by liquid chromatography-tandem mass spectrometry (LC-MS/MS). Liver proteins were analyzed by using an Orbitrap Astral mass spectrometer. Colon content microbiota composition was assessed via metagenomics. Signaling pathway protein expression was analyzed via Western blotting (WB). **Results**: SPNSs significantly increased the red blood cell (RBC) count and hemoglobin (HGB) level by day 14 and alleviated hepatointestinal damage. Hepatic metabolomics revealed: the most abundant metabolites were fatty acids and stachyose on day 7 and amino acid and arachidonic acid derivatives on day 14. KEGG analysis implicated cAMP signaling. Proteomics revealed upregulated immune-related proteins and enhanced PI3K pathway activity (WB-validated). Colon content metabolomics showed increased daidzein, 3-(2,5-dimethoxyphenyl) propanoic acid, γ-CEHC, and adenosine in SPNS groups on day 14. Metagenomics indicated differential abundances of *Heminiphilus faecis*, *Phocaeicola sartorii*, and *s-bacterium_J10.2018* on day 14. Multiomics integration demonstrated significant correlations between hepatic metabolites, hematopoietic proteins, colon content metabolites, and probiotic bacteria. **Conclusions:** SPNS alleviates cyclophosphamide-induced hepato-intestinal injury in anemic mice by modulating the gut microbiota and enhancing hepato-intestinal immune defense. Additionally, SPNSs ameliorate anemia in cyclophosphamide-treated mice by activating the cAMP/PI3K/AKT pathway, promoting hepatocyte proliferation, and increasing hematopoietic protein expression.

## 1. Introduction

Cyclophosphamide (CTX), an immunosuppressive agent, suppresses bone marrow function and inhibits hematopoiesis [1]. Concurrently, CTX depletes hepatic glutathione levels, inducing oxidative stress and liver injury [2]. Liver damage is also recognized as a crucial factor contributing to hematopoietic system diseases. Some patients with hepatitis may develop at least one of the following conditions: anemia, thrombocytopenia, or neutropenia [3]. Brandon J reported that autoimmune hepatitis can cause severe anemia [4]. These findings suggest a potential close relationship between liver changes and anemia in cyclophosphamide-treated patients. Clinically, CTX therapy can also adversely affect digestive function, leading to symptoms such as vomiting and loss of appetite. Therefore, CTX can induce hepato-intestinal injury, thereby causing anemia.

*Panax notoginseng* (PN) is one of the major herbal plants in the genus Panax (Araliaceae). It can have beneficial effects on blood cells, and its main active ingredients are rare saponins. Steamed *Panax notoginseng* saponins (SPNSs) contain rare saponins that alleviate CTX-induced anemia [5]. Orally administered steamed *Panax notoginseng* powder or SPNSs may modulate the gut microbiota composition and metabolite profile. Through bidirectional interactions, saponin-derived metabolites enter the circulation via the intestinal mucosa to influence distant organs; gut microbes metabolize host-derived compounds into bioactive secondary metabolites; and the microbiota directly utilizes metabolites for growth, altering the microbial composition [6]. Complex interactions between them can be discovered through the study of metabolomics and gut microbiota diversity [7].

Metagenomics, also known as microbial environmental genomics, can comprehensively and precisely display the overall functional metabolic spectrum and species composition of microbial communities and clarify the fundamental mechanism by which microbial communities play a role in ecosystems in principle. The effects of *Bifidobacterium* subspecies on functional dyspepsia were reported through metagenomic and metabolomic analyses [8]. It has also been reported that Rk_3_ can restore gut microbiota homeostasis and significantly increase the level of the microbial metabolite butyrate. By inhibiting histone deacetylase (HDAC) activity and activating the JAK/STAT3 signaling pathway, it thereby protects dopaminergic neurons and alleviates neuroinflammation [9]. Up- and down-regulation of the gut microbiota and their metabolites are key factors in the health impacts linked to dietary protein [10]. The potential connections among the three can be revealed by analyzing the relationships among the gut microbiota, metabolism, and health [11].

We previously developed an optimized steaming protocol for the production of SPN enriched with rare saponins that effectively ameliorate CTX-induced anemia [12]. Nevertheless, the effects of SPNSs on intestinal microenvironment remodeling and hepatic functional recovery and the role of SPNSs in ameliorating cyclophosphamide-induced anemia remain unclear. The interaction between the liver and the intestine in terms of metabolism and the microbiota is called the liver-intestinal or hepatogut axis [13]. This study aimed to elucidate the effects of SPNSs on the intestinal flora, metabolite profiles and cyclophosphamide-induced anemia from the perspective of the hepatogut axis through nontargeted metabolomics, proteomics and metagenomics. It is widely acknowledged that anemia can pose a serious threat to human health and significantly compromise the recovery of patients undergoing CTX therapy. This research seeks to provide valuable insights for subsequent clinical research and application.

## 2. Materials and Methods

### 2.1. Main Reagents

SPNSs (83.8% saponin content) were prepared in-house. The concentrations of saponins were detected by high-performance liquid chromatography (HPLC). Cyclophosphamide (CTX) and acetylphenylhydrazine (APH) were obtained from Shanghai Aladdin Biochemical Technology (Shanghai, China). Erythropoietin (EPO) was sourced from Chengdu Di’ao Jiuhong Pharmaceutical (Chengdu, China). Western blot membrane stripping buffer was obtained from Solarbio (Beijing, China).

### 2.2. Animal Ethics

Male KM mice were purchased from SPF (Beijing) Biotechnology Co., Ltd. (Beijing, China) and housed in the animal facility of Kunming University of Science and Technology. The environmental conditions were maintained at 24 °C and 65% humidity. All procedures were approved by the Kunming University Institutional Animal Care and Use Committee (Approval No. PZWH (Dian) K2024-0013; Date: 31 May 2024) and strictly followed the university’s Guidelines for the Care and Use of Laboratory Animals.

### 2.3. Animal Experiments

Ninety-six male specific pathogen-free (SPF)-grade KM mice (20 ± 2 g) were acclimatized for one week and randomly assigned to six groups (*n* = 16/group): the control, model (CTX), positive control (EPO), low-dose SPNS (SPNS20), medium-dose SPNS (SPNS40), and high-dose SPNS (SPNS80) groups. The mice in all the groups except those in the control group received intraperitoneal CTX (40 mg/kg/day) for 4 days. On days 1 and 4, these mice were also injected with APH (20 mg/kg and 40 mg/kg, respectively) to induce anemia. Beginning on day 5, the mice were fasted for 4 h before daily treatments. The control and CTX groups received saline via oral gavage daily. The EPO group received subcutaneous EPO (1500 IU/kg) every other day [14]. The SPNS groups received daily oral SPNS at 20, 40, or 80 mg/kg [15]. Body weight was recorded every other day throughout the study.

### 2.4. Anemia Parameter Assessment

After 7 and 14 days of treatment, approximately 0.5 mL of blood was collected via retro-orbital puncture into anticoagulant tubes. Red blood cell (RBC) counts and hemoglobin (HGB) concentrations were obtained using a Mindray veterinary automated hematology analyzer.

### 2.5. Histological Analysis

Liver and small intestine tissues were fixed in 10% formalin buffer, paraffin-embedded, and sectioned. Then, 5 μm thick sections were deparaffinized, rehydrated, and stained with hematoxylin and eosin (HE). Images were captured using an Olympus light microscope at 100× and 400× magnification.

### 2.6. Liver Metabolomics

Liver tissues collected on days 7 and 14 were snap-frozen in liquid nitrogen and stored at −80 °C. Metabolites were extracted and analyzed by liquid chromatography tandem mass spectrometry (LC-MS/MS). The raw data were processed using Progenesis QI (Waters Corporation, Milford, CT, USA). Metabolite identification was performed with public databases for HMDB (http://www.hmdb.ca/) and Metlin (https://metlin.scripps.edu/) and the Majorbio in-house library. Differentially abundant metabolites were identified (VIP > 1, *p* < 0.05) and visualized using R packages (Version 1.6.2).

### 2.7. Liver Proteomics

Liver tissues harvested on day 14 were processed for proteomics. Proteins were extracted, quantified, digested, and desalted. Peptides were separated by HPLC (Vanquish Neo system) and analyzed by DIA-MS using an Orbitrap Astral mass spectrometer (positive ion mode; source voltage: 1.5 kV; scan range: *m*/*z* 100–1700). The data were analyzed on the Majorbio Cloud Platform (https://cloud.majorbio.com (accessed on 17 August 2025). Differentially expressed proteins were defined as those with a fold change > 1.2 and *p* < 0.05 (Student’s *t* test in R).

### 2.8. Western Blotting

Proteins (40 μg) were separated by 10% SDS-PAGE and transferred to PVDF membranes. The membranes were blocked with 5% skim milk (2 h, 37 °C), incubated with primary antibodies overnight (4 °C), washed with Tris-buffered saline containing Tween-20 (TBST), and incubated with secondary antibodies (2 h, RT). The specific primary antibodies used were anti-PI3K (CST, USA,#4249, 1:1000), anti-ERK (CST, USA #4695, 1:1000), anti-phosphorylated ERK (Wanleibio, Shengyang, China, #WLP1512, 1:500), anti-CREB (CST, USA, #9197, 1:1000), anti-phosphorylated CREB (Abcam, UK #AB81293, 1:1000), anti-AKT (Abmart, Shanghai, China #T55561S, 1:500), anti-phosphorylated AKT (Proteintech, Wuhan, China, #28731-1-AP,1:1000), and anti-β-actin (Proteintech, Wuhan, China, #20536-1-AP, 1:5000). The secondary antibodies used were Horseradish Peroxidase (HRP) Goat Anti-Rabbit lgG (H + L) Antibody (K1223, 1:5000) from Apexbio (Beijing, China). Proteins were detected using chemiluminescence. The membranes were stripped and reprobed for different targets using stripping buffer. Band intensity was quantified using ImageJ software (version ImageJ 1.53e).

### 2.9. Colon Content Metabolomic Analysis

Colon contents collected aseptically on days 7 and 14 were snap-frozen, stored at −80 °C, and shipped on dry ice. Metabolites were extracted with isotope-labeled internal standards. A Waters ACQUITY UPLC BEH amide column on a Vanquish UHPLC system was used for separation. Mass spectrometry was performed using an Orbitrap Exploris 120 (Xcalibur 4.4 software). The raw data (converted to mzXML via ProteoWizard) were annotated against BiotreeDB (V3.0) and visualized with R (Version 1.0.12).

### 2.10. Colon Content Metagenomic Analysis

Colon contents (on days 7 and 14) were collected aseptically, frozen, and stored as described above. Microbial DNA was extracted, quantified, and used for library preparation. Sequencing (PE150) was performed on an Illumina NovaSeq 6000 platform. Quality-filtered reads were assembled (MEGAHIT v1.2.9). Contigs underwent CDS prediction (MetaGeneMark v3.26), clustering, and redundancy removal (CD-HIT v4.6.1). TPM-normalized abundances were calculated. Unigenes were annotated against the NR database for taxonomic profiling. Species-level abundance and differential analyses were conducted.

### 2.11. Multiomic Integration

Spearman correlation analysis was performed between the liver metabolome and proteome (day 14), liver and colon content metabolomes (day 14), and the colon content metabolome and colon content metagenomics (days 7 and 14) in the CTX and SPNS groups. Correlations were computed using the cor.test function in R, generating matrices of correlation coefficients (Corr) and *p* values. Significant associations (*p* < 0.05) were visualized.

### 2.12. Statistical Analysis

The data are presented as the means ± standard deviations (SDs). Statistical significance was determined using one-way ANOVA followed by appropriate post hoc tests in GraphPad Prism 10.0.2. Differences were considered significant at * *p* < 0.05 and ** *p* < 0.01.

## 3. Results

### 3.1. Effects of SPNS on Body Weight and Anemia Parameters in CTX-Induced Anemic Mice

**Figure 1:**
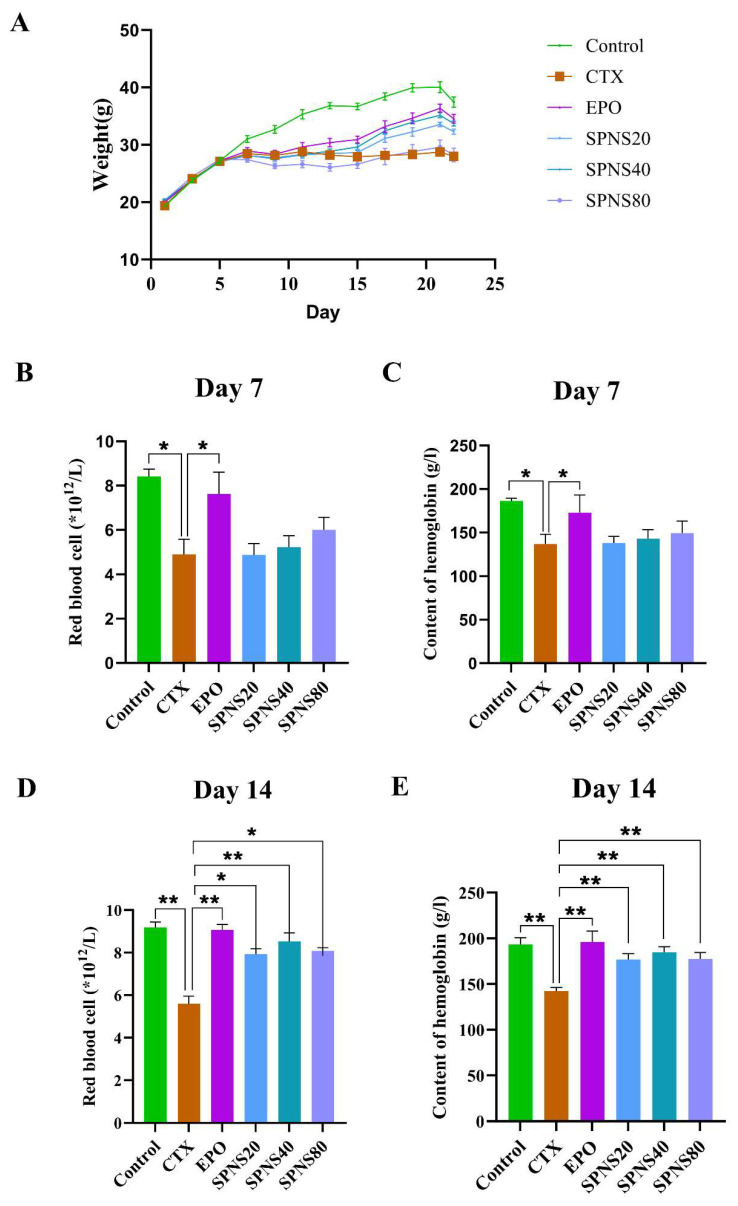
Body weight changes in the mice and comparisons of blood parameters associated with anemia. (**A**) Body weight changes in the mice. (**B**) RBC counts on day 7. (**C**) Levels of HGB on day 7. (**D**) RBC counts on day 14. (**E**) HGB levels on day 14. Each value represents the mean ± SD (*n* = 6); * *p* < 0.05 and ** *p* < 0.01.

Body weight monitoring revealed consistent daily gains (~2 g) across all groups during acclimatization. Following CTX administration, weight gain plateaued or declined. The EPO, SPNS20 and SPNS40 groups exhibited gradual weight recovery, whereas the SPNS80 group presented no significant increase in body weight (Figure 1A). RBC and HGB analyses confirmed successful anemia induction, with the CTX group showing significantly lower levels than the control group on day 7. The SPNS (20/40/80) groups presented dose-dependent increases in RBC count and HGB level, although these increases did not reach statistical significance (*p* > 0.05) compared with those of the CTX group on day 7, unlike those of the EPO group (*p* < 0.05) (Figure 1B,C). By day 14, all the treatment groups presented significantly greater RBC counts than the CTX group (*p* < 0.01 for the EPO and SPNS40 groups; *p* < 0.05 for the SPNS20 and SPN80 groups). HGB levels were significantly elevated in all treatment groups compared with those in the CTX group on day 14 (*p* < 0.01) (Figure 1D,E).

### 3.2. Ameliorative Effects of SPNS on CTX-Induced Histopathological Injury in the Liver and Small Intestine of Anemic Mice

**Figure 2:**
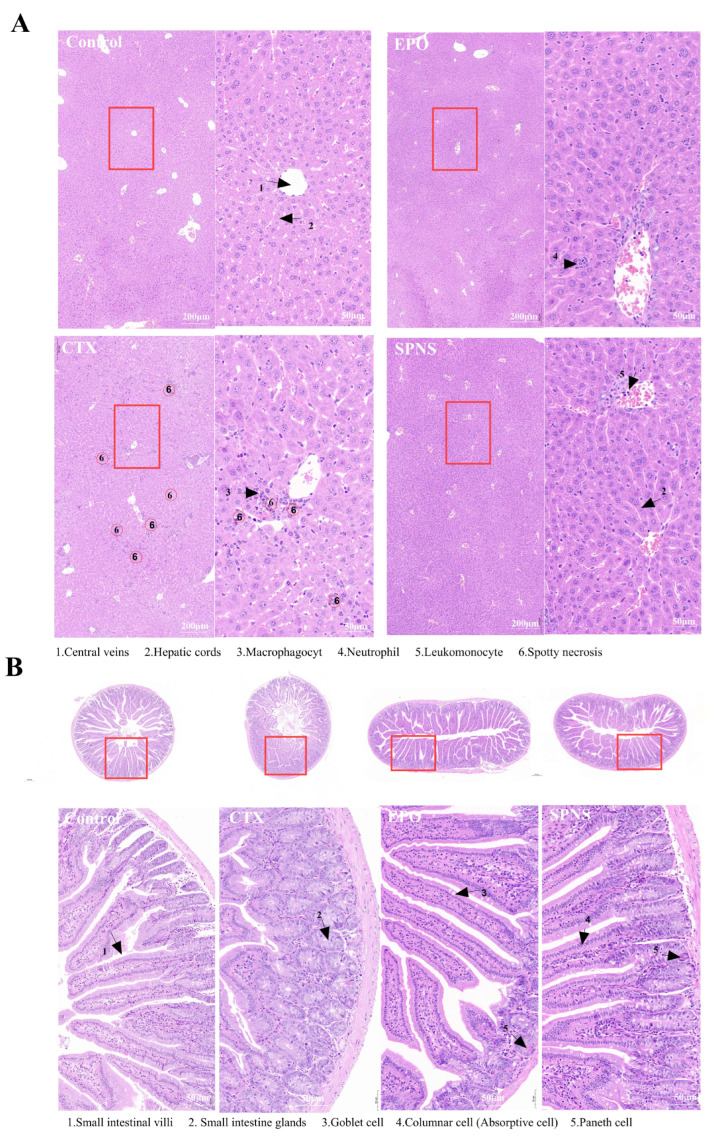
Histopathological staining. (**A**) HE staining of the liver tissue and (**B**) HE staining of the small intestine.

Liver histology (Figure 2A): The CTX group exhibited multifocal punctate necrosis, disorganized hepatocyte arrangement, disrupted hepatic cords, marked inflammatory cell infiltration, and central vein distortion or absence. The EPO and SPNS groups presented regenerating hepatocytes with orderly arrangements, distinct hepatic cords, and minimal inflammation. Small intestine histological analysis (Figure 2B) revealed that the CTX group presented with villus fusion, architectural distortion, reduced goblet cells, Paneth cell degeneration and small intestinal gland hyperplasia. The control, EPO, and SPNS groups maintained slender, well-aligned villi with preserved architecture, increased goblet cells, and clearly identifiable Paneth cells in the crypts.

### 3.3. Analysis of Differential Metabolites and Pathway Enrichment in Liver Tissue of the CTX and SPNS Groups

**Figure 3:**
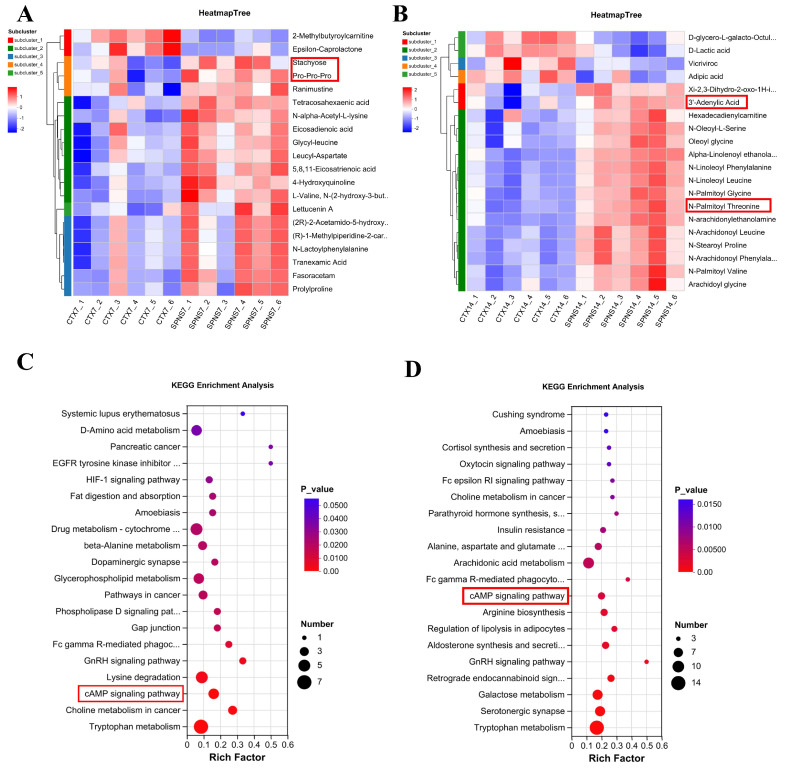
Metabolomic analysis of hepatic-tissues from the SPNS vs. CTX groups. (**A**,**B**) Clustering heatmaps of the top 20 differentially abundant metabolites at days 7 and 14. (**C**,**D**) KEGG enrichment bubble plot at days 7 and 14. The red frame highlights the metabolites and signaling pathways of particular interest.

Principal component analysis (PCA) demonstrated high intragroup reproducibility of the samples collected on days 7 and 14 (the CTX and SPNS groups) and clear intergroup separation in the CTX vs. SPNS samples collected on day 14 (Figure S1a,b). We identified 5108 and 5311 metabolites in the samples collected on days 7 and 14, respectively. Compared with the CTX group, the SPNS group presented 816 differentially abundant metabolites on day 7 (751 increased, 65 decreased; Figure S1c) and 1393 on day 14 (298 increased, 1095 decreased; Figure S1d). The top 20 differentially abundant metabolites were observed as follows: On day 7, the abundance of stachyose, etc., was increased in the SPNS group (Figure 3A). On day 14, the abundance of 3′-Adenylic Acid, *N*-Palmiloy Threonine, etc., was significantly increased (Figure 3B). We performed a comparative KEGG enrichment analysis of the top 20 differential pathways between the SPNS and CTX groups. The results, presented in a bubble plot, revealed that the cAMP signaling pathway was significantly upregulated at both time points (day 7 and day 14) (Figure 3C,D).

### 3.4. Analysis of Differential Proteins and Functional Enrichment in Liver Tissues of the SPNS and CTX Groups

**Figure 4:**
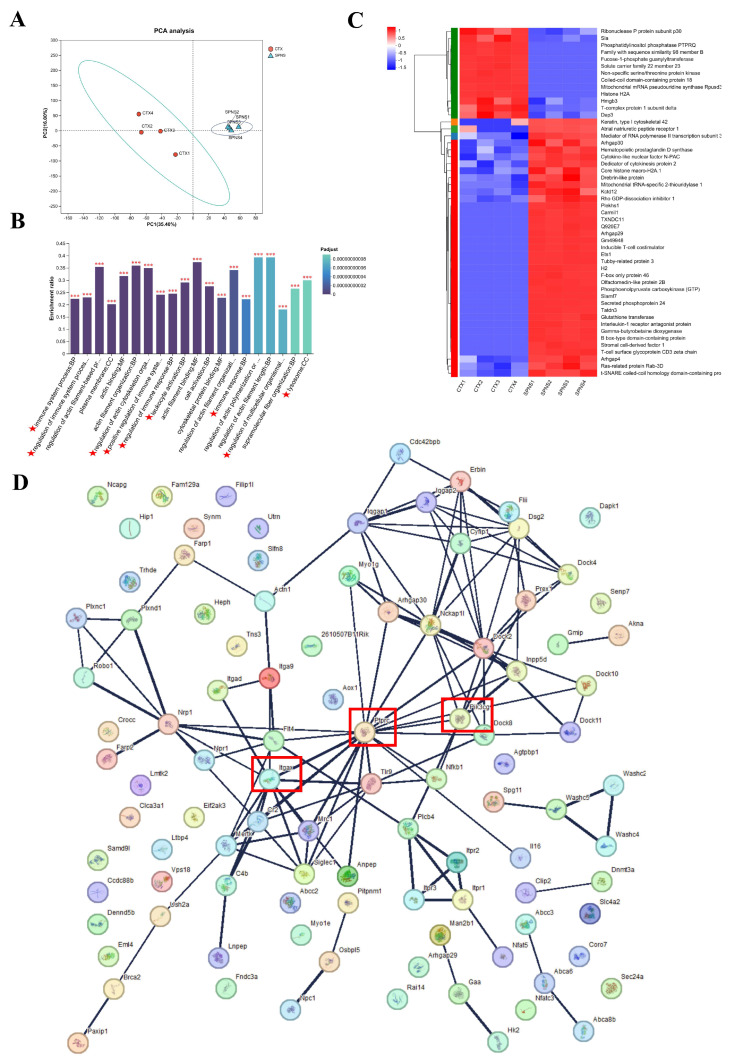
Proteomic analysis of hepatic tissue. (**A**) PCA of samples. (**B**) GO enrichment analysis of the SPNS vs. CTX groups. (**C**) Cluster heatmap of the top 50 differentially expressed proteins. (**D**) Protein-protein interaction network of the SPNS vs. CTX groups. The red stars indicate the GO functional enrichment of immune-related proteins. The red frame highlights the key node proteins. Differences were considered significant at *** *p* < 0.001.

PCA revealed distinct separation between the SPNS and CTX groups and high intragroup homogeneity (Figure 4A). GO enrichment analysis of the top 20 upregulated proteins in the SPNS vs. CTX comparison highlighted immune processes, including the immune response, regulation, and lymphocyte activation (Figure 4B). Venn analysis revealed 46 overlapping proteins between the SPNS-CTX group differential proteome and the PI3K signaling pathway (Figure S2). Heatmap clustering of the top 50 differentially expressed proteins revealed 37 upregulated and 13 downregulated proteins in the SPNS group compared with the CTX group (Figure 4C). Protein-protein interaction network analysis revealed key hub proteins (ItgaX, Ptprc, Pik3cg) within the SPNS-CTX group differential proteome (Figure 4D).

### 3.5. The Effect of SPNS on the Expression of Proteins Related to the cAMP/PI3K/AKT Signaling Pathway

**Figure 5:**
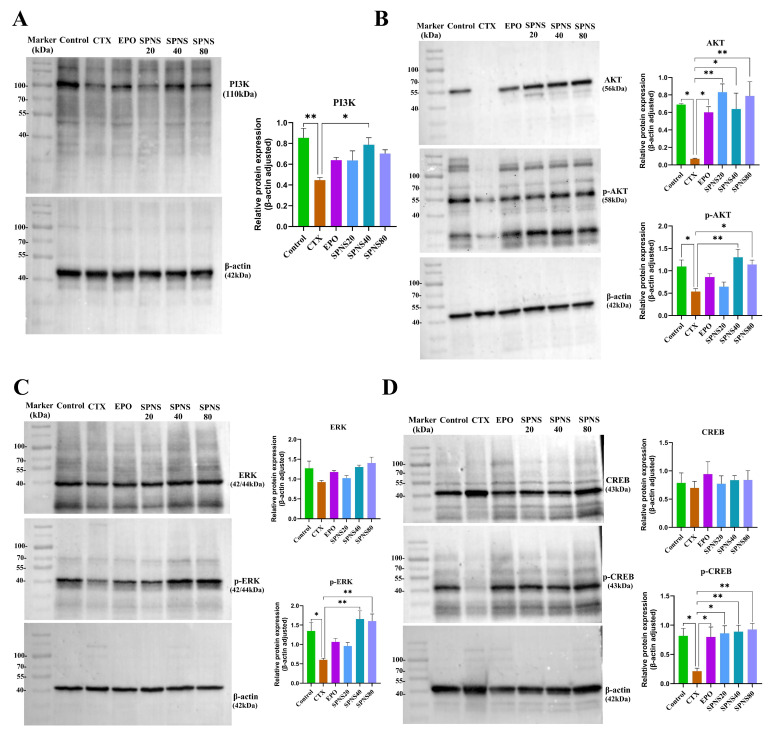
Relative expression levels of key proteins in hepatocyte proliferation signaling pathways. (**A**) Relative expression level of PI3K (p110). (**B**) Relative expression levels of AKT and *p*-AKT. (**C**) Relative expression levels of ERK and *p*-ERK. (**D**) Relative expression levels of CREB and *p*-CREB. Each value represents the mean ± SD (*n* = 3–4); * *p* < 0.05 and ** *p* < 0.01.

Based on the metabolomics results, showing involvement of the PI3K/AKT and cAMP signaling pathways (linked to proliferation/survival), we validated key proteins by Western blotting (Figure 5). Compared with those in the CTX group, the relative expression levels of PI3K (p110) were significantly greater in the control (*p* < 0.01) and SPNS40 groups (*p* < 0.05). The other groups presented nonsignificant increases (Figure 5A). AKT was significantly elevated in the SPNS20 and SPNS80 groups (*p* < 0.01) and in the control, EPO and SPNS40 groups (*p* < 0.05) compared with the CTX group. *p*-AKT (Ser473) was significantly greater in the SPNS40 group (*p* < 0.01) and in the control and SPNS80 groups (*p* < 0.05) compared to the CTX group, and the other groups were not significantly changed (Figure 5B). The relative expression levels of ERK and CREB also did not significantly change. *p*-ERK and *p*-CREB levels were significantly greater in the SPNS40 and SPNS80 groups than in the CTX group (*p* < 0.01; Figure 5C,D).

### 3.6. Comparative Analysis of the Effects of SPNS on Colon Metabolites in CTX-Induced Anemic Mice

**Figure 6:**
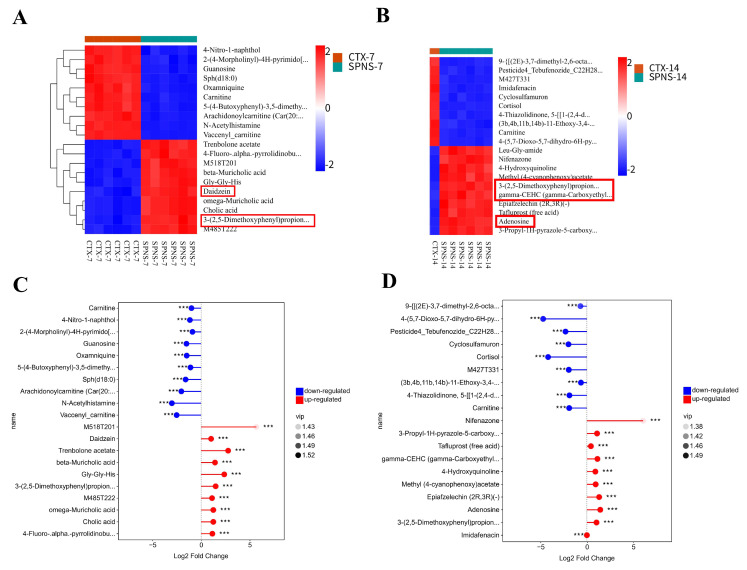
Metabolomic analysis of colon contents. (**A**,**B**) Heatmap of the top 20 differentially abundant metabolites. (**C**,**D**) Multiple differences in the abundances of metabolites between the CTX and SPNS groups were detected on days 7 and 14, respectively. Differences were considered significant at *** *p* < 0.001). The red frame highlights the metabolites of particular interest.

We identified 6489 colon metabolites. PCA confirmed good reproducibility among the samples collected on days 7 and 14 (Figure S3a,b). The metabolites were classified as benzene derivatives, organic acids/derivatives, lipids/lipid-like molecules, organoheterocyclic compounds, or amino acids (Figure S3c). The top 20 differentially abundant metabolites (SPNS vs. CTX) acted as follows: On day 7, the SPNS group presented significantly increased abundances of metabolites such as 3-(2,5-dimethoxyphenyl) propanoic acid, daidzein, etc. (Figure 6A). On day 14, the abundances of adenosine, 3-(2,5-dimethoxyphenyl) propanoic acid, carboxyl-hydroxytryptophine (γ-CEHC), etc., were increased (Figure 6B). The fold changes in the significantly differentially abundant metabolites are shown in Figure 6C,D.

### 3.7. Analysis of Colon Content Microbiota Differences Between the SPNS Group and the CTX Group

**Figure 7:**
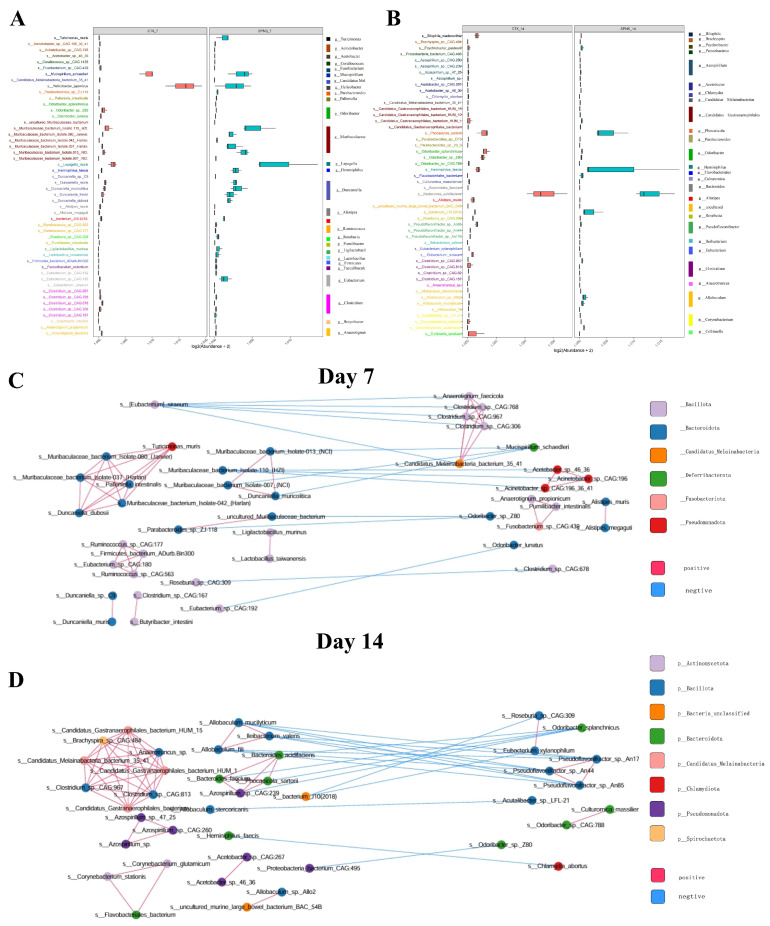
Colon bacterial characteristics of the CTX and SPNS groups at days 7 and 14, respectively. (**A**,**B**) Relative abundances of 50 bacterial species responsible for discriminating between the CTX and SPNS groups. Co-occurrence network deduced from bacterial taxa differentially enriched in the CTX and SPNS groups. The co-occurrence network of day 7 (**C**) and day 14 (**D**). Red edges indicate positive correlations, and blue edges indicate negative correlations.

Principal coordinate analysis (PCoA) revealed distinct clustering between the CTX and SPNS groups at days 7 and 14 (Figure S4a,c). The alpha diversity (species level) was greater in the SPNS group than in the CTX group, although the difference was not statistically significant (Figure S4b,d). The top 50 differential bacterial species on days 7 and 14 are shown, respectively (Figure 7A,B). The key bacterial genera and significantly different species are summarized in Table 1. Co-occurrence networks of differentially abundant species revealed strong correlations among bacteria within the same genus at both day 7 and day 14. For example, we observed that species belonging to the genera Muribaculaceae_bacterium, Turicimonas_muris, and Duncaniella_dubosii were positively correlated and constituted a symbiotic bacterial community on day 7 (Figure 7C). Furthermore, several Candidatus species and Clostridium-enriched species were closely correlated to generate a covarying cluster on day 14 (Figure 7D).

### 3.8. Correlation Analysis: Hepatic Metabolites, Proteins, Colon Metabolites, and Colon Microbiota

**Figure 8:**
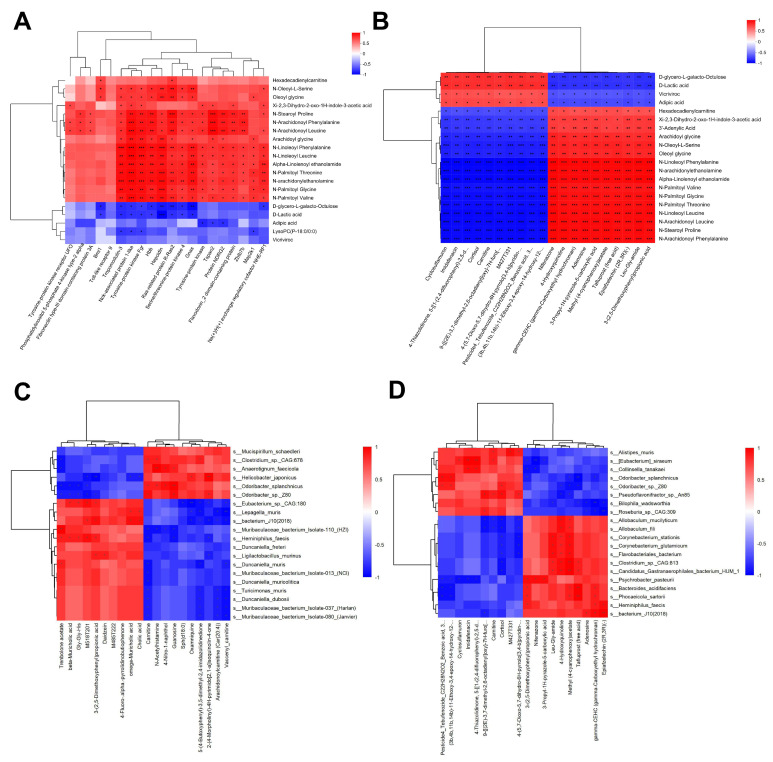
Multiomics correlation analysis. (**A**) Correlation analysis of liver metabolite abundance and liver protein levels. (**B**) Correlation analysis of the abundances of liver metabolites and colon metabolites. (**C**) Correlation analysis of the abundances of colon metabolites and colon bacteria on day 7. (**D**) Correlation analysis of the abundances of colon metabolites and colon bacteria on day 14. Differences were considered significant at * *p* < 0.05, ** *p* < 0.01 and *** *p* < 0.001.

Spearman correlation (the SPNS group vs. the CTX group): On day 14, the abundance of 15 of the top 20 hepatic metabolites was positively correlated with hematopoietic-related protein levels (Figure 8A). The proteins with the strongest correlations included protocadherin, NCKAP1, Fgr tyrosine kinase, hemoglobin subunit beta, hemojuvelin, and RRAS2. Analysis revealed significant correlations between the top 20 most abundant hepatic metabolites and colon metabolites (Figure 8B). We identified 30 gut metabolite–bacteria pairs that were significantly positively correlated (*p <* 0.05) and 4 pairs that were highly significantly correlated (*p <* 0.01) on day 7, and 50 pairs (*p <* 0.05) and 7 pairs (*p <* 0.01) on day 14 between the gut metabolites and bacterial species (Figure 8C,D).

## 4. Discussion

The relationships between diseases and the liver and intestines have attracted increasing attention. The intestinal microbiota also plays a crucial role in the digestion and metabolism of substances. As well as establishing homeostasis in the intestines and liver, metabolites, microorganisms, and immune system regulation are extremely important for health. CTX compromises intestinal mucosal integrity and barrier function [16], potentially inducing dysbiosis and immune dysregulation [17]. As mentioned earlier, CTX can cause liver damage. Liver and intestinal damage are likely to affect the digestion, absorption, and metabolism of substances, as well as the expression of proteins.

Our research constructed an animal model of anemia using the immunosuppressive effect of CTX. The experimental results revealed that CTX caused hepatointestinal damage, and the weight of the mice stopped increasing. After SPNS treatment, the body weights of the mice gradually increased, which might be related to the repair of hepatoenteric cells and structures. The results of HE staining also indicated that clear Paneth cells were visible at the base of the small intestine in the SPNS group. These cells secrete immune molecules such as defensins, antimicrobial peptides, lysozymes and growth factors to mediate innate immunity and participate in the regulation of the intestinal flora [18].

Some upregulated, differentially abundant metabolites were detected in the hepatic metabolites on day 7 in the SPNS group. Stachyose can alleviate the intestinal mucosal injury in mice induced by cyclophosphamide by regulating the miRNAs in intestinal exosomes [19]. Poly-L-lysine plays a role in gastrointestinal inflammatory diseases [20]. Tetracosahexaenoic acid is a type of polyunsaturated fatty acid that belongs to the omega-3 fatty acid group. It plays a role in oxidative stress and immune regulation [21]. By day 14, 3′-Adenylic acid is used for the synthesis of cyclic adenosine monophosphate (cAMP) (a precursor for cAMP signaling) [22]. *N*-Palmitoyl threonine is associated with alleviating intestinal mucosal damage [23]. This metabolic reprogramming suggests that SPNSs facilitate gut–liver repair.

Hepatic proteomics revealed 37 upregulated proteins in the SPNS group. These include regulators of proliferation/differentiation (Arhgap30, Arhgap29, Dedicator of cytokinesis protein 2, etc.) [24] and cell cycle/signaling modulators (Plekhs1, Tubby-related protein 3, Ras-related protein Rab-3D, etc.). TAMARA A M C reported that Plekhs1 could increase the phosphorylation of PI3K and src family kina-dependent Y (258) XXM, thereby triggering PI3K activation [25]. The levels of immune regulators (inducible T cells, Ets1, Slamf7) [26], antioxidant proteins (hematopoietic prostaglandin D synthase, glutathione transferase, interleukin-1 receptor antagonist protein) [27], and tissue repair factors (Tatdn3, stromal cell-derived factor 1, etc.) [28] were also elevated. GO enrichment analysis confirmed that SPNS enhances hepatic immune responsiveness and regulation.

**Figure 9:**
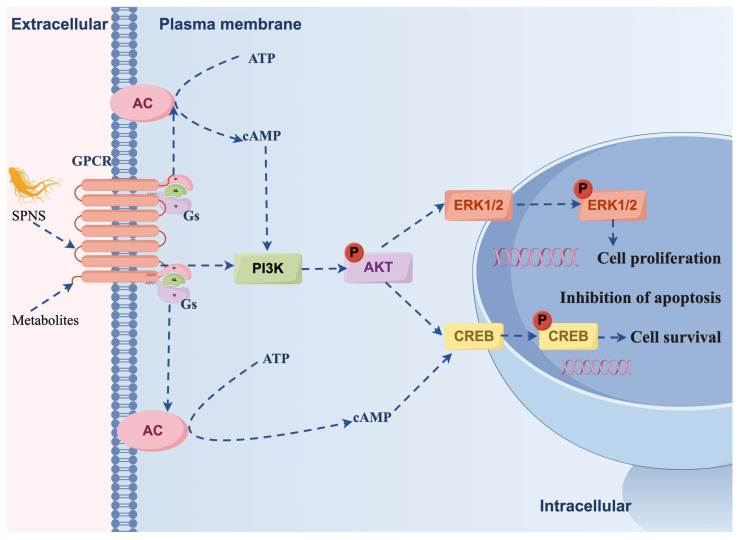
The cAMP/PI3K/AKT signaling pathway promotes hepatocyte proliferation and survival.

The differential protein interaction network between the SPNS and CTX groups revealed important node proteins: ItgaX is a member of the integrin family, Ptprc is the protein tyrosine phosphatase receptor C, and there are also other proteins involved. These proteins are closely related to the PI3K/AKT signaling pathway [29,30]. Taken together, these findings indicate that SPNS promotes hepatocyte proliferation/survival, potentially via cAMP and PI3K/AKT pathway activation (Figure 9). Western blotting and histology confirmed that PI3K (p110), AKT, *p*-AKT, *p*-ERK, and *p*-CREB were upregulated alongside liver structural recovery. However, it is important to note that direct evidence of whether SPNS regulates hepatocyte proliferation and survival through activation of the cAMP/PI3K/AKT pathway has not yet been established. We plan to inhibit the cAMP, PI3K, and AKT pathways using Rp-cAMPS, LY294002, and MK-2206, respectively, in our subsequent studies. The phosphorylation levels of key proteins, such as *p*-AKT (Ser473), total AKT, and downstream targets, will then be examined by Western Blot analysis. This is crucial for strengthening the conclusions of our study and for further mechanistic investigations.

The liver is the site at which many proteins are produced. Hematopoietic-related proteins were enriched through liver proteomics. Among these proteins, HBB is the B subunit of hemoglobin and jointly completes the synthesis of hemoglobin with HBA. Hepcidin not only participates in iron metabolism but also has antibacterial functions [31]. Tropomodulin is a protein that binds to both red blood cells and non-red blood cells and covers the tips of actin filaments. Nck-associated protein 1-like, serine/threonine-protein kinase 4 (PAK4), etc., play a role in signal transduction and cell survival. A correlation analysis of the top 20 metabolites revealed a significant positive correlation, indicating that these metabolites play a positive role in the process of hematopoietic protein participation in hematopoiesis.

Metabolomic analysis of the colon contents revealed upregulated metabolites in the SPNS group on day 7. Daidzein can activate the PI3K/AKT/CREB signaling pathway, inhibit apoptosis, protect mucosal integrity, and alleviate gastrointestinal injury [32]. These findings, combined with the results of liver metabolomics on day 7, indicate that SPNS treatment initiates the defense against and repair of the damaged intestinal tract in mice at an early stage. The metabolites significantly upregulated on day 14 included γ-CEHC, adenosine, and 3-(2,5-dimethoxyphenyl) propionic acid. γ-CEHC, a cytochrome P450-derived metabolite, exhibits natriuretic activity and possesses anti-inflammatory properties. Adenosine mediates critical functions in energy metabolism, cellular signaling, and immune regulation. 3-(2,5-Dimethoxyphenyl) propionic acid was significantly increased in the PNS groups at both days 7 and 14, suggesting its potential utility as a biomarker.

Metagenomic analysis of the colon contents revealed that SPNS altered the abundance of colon bacterial species and the structure of the microbiota. Compared with those in the CTX group, the number of differentially expressed colon bacterial species changed in the SPNS group at days 7 and 14. The abundances of *Heminiphilus_faecis*, *Phocaeicola_sartorii* and *s-bacterium_J10.2018* significantly increased in the SPNS group on day 14. *Heminiphilus_faecis* and *Phocaeicola_sartorii* are important intestinal probiotics [33,34]. The analysis of colon metabolites revealed that they were significantly positively correlated with γ-cehc. *Heminiphilus_faecis* and *Phocaeicola_sartorii* were significantly positively correlated with 3-(2,5-dimethoxyphenyl). These metabolites also had a significant positive correlation with the key metabolites of the liver. Therefore, *Heminiphilus_faecis*, *Phocaeicola_sartorii* and *s-bacterium_J10.2018* may play important roles in regulating intestinal metabolism and liver metabolism.

This study has several limitations that should be considered. First, our experiments were conducted exclusively on male mice, which precludes the examination of any sex-specific effects [35]. Second, the experimental period may not have been sufficient to observe long-term effects, leaving the long-term outcomes unexplored. Furthermore, the sample size for the molecular assays was small, potentially limiting the statistical power of these specific analyses. Finally, translating these preclinical findings from mice to human applications presents significant challenges, and thus the clinical relevance still needs to be established.

In summary, this study reveals that the SPNS constructs an intestinal–liver–hematopoietic network that multi-dimensionally improves the anemia induced by CTX. The finding holds significant promise for addressing a persistent and debilitating challenge in clinical oncology. The most direct translational implication is the potential development of SPNS or its active components as an adjuvant therapy. A natural product-based intervention like SPNS could offer a complementary or alternative strategy, potentially with a more favorable safety profile. Future research should focus on isolating the specific bioactive compounds within SPNS responsible for the hematoprotective effect, which could be developed into a standardized, pharmaceutical-grade supplement for patients undergoing myelosuppressive chemotherapy.

## 5. Conclusions

SPNS alleviates cyclophosphamide-induced hepato-intestinal injury in anemic mice by modulating the intestinal microbiota and enhancing hepato-intestinal immune defense. Additionally, SPNSs ameliorate anemia in cyclophosphamide-treated mice by activating the cAMP/PI3K/AKT pathway, promoting hepatocyte proliferation, and increasing hematopoietic protein expression. However, it is still unclear how the saponin components in SPNS activate the cAMP/PI3K/AKT signaling pathway. Future studies will further investigate the mechanisms and validate the findings in clinical settings.

## Figures and Tables

**Table 1:** Significantly different bacterial genera and species in SPNS vs. CTX groups.

	Featured Bacterial Genera	Significantly Different Bacterial Species
day 7	Muribaculaceae (7 species), Duncaniella (5 species), Odoribacter (3 species)	*Lepagella muris, Muribaculaceae bacterium Isolate-110-HZI, Mucispirillum schaedleri, Duncaniella muricolitica, Eubacterium sp. CAG-180, Turicimonas muris*
day 14	Azospirillum, Allobaculum, Clostridium, Candidatus Gastranaerophilales (4 species), Pseudoflavonifractor, doribacter, Corynebacterium (3 species)	*Heminiphilus faecis, Phocaeicola sartorii, and s-bacterium_J10.2018*

## Data Availability

The data presented in this study are available on request from the corresponding author due to privacy.

## Supplementary Materials

The following supporting information can be downloaded at: https://www.mdpi.com/article/10.3390/nu17213335/s1. Figure S1. Metabolomic analysis of hepatic tissues. (a,b) PCA of the samples at days 7 and 14. (c,d) Histogram of differentially abundant metabolites among the groups of samples. Figure S2. Proteomic analysis of hepatic tissue. Venn diagram of the differential protein sets between the SPNS and CTX groups and the protein sets of the PI3K signaling pathway. Figure S3. Metabolomic analysis of intestinal contents. (a,b) PCA of samples. (c) Metabolite classification pie chart. Figure S4. Gut bacterial characteristics of the CTX and SPNS groups at days 7 and 14, respectively. (a,c) PCA of samples. (b,d) Comparison of gut bacterial α diversity between the CTX and SPNS groups (Wilcoxon rank sum test).

## Abbreviations

AKT, protein kinase B; APH, Acetyl phenyl hydrazine; cAMP, cyclic adenosine monophosphate; CREB, cAMP-response element binding protein; CTX, Cyclophosphamide; EPO, Erythropoietin; ERK, Extracellular signal regulated kinase; HE, Hematoxylin and eosin; vs, versus; HGB, Hemoglobin; 7d, day 7; 14d, day 14; HPLC, High-performance liquid chromatography; LC-MS, Liquid chromatography tandem mass spectrometry; *p*-AKT, Phosphorylated protein kinase B; *p*-CREB, Phosphorylated cAMP-response element binding protein; *p*-ERK, Phosphorylated Extracellular signal regulated kinase; PI3K, Phosphoinositide 3-kinase; RBC, Red blood cell; SPNS, Steamed Panax notoginseng Saponins; TBST, Tris-buffered saline containing Tween-20; SD, standard deviation; WB, Western Blot.
