# Supplementary material for: Steamed Panax notoginseng Saponins Ameliorate Cyclophosphamide-Induced Anemia by Attenuating Gut-Liver Injury and Activating the cAMP/PI3K/AKT Signaling Pathway
Source: Nutrients. 2025 Oct 23;17(21):3335. doi: 10.3390/nu17213335 (PMC12608177; doi:10.3390/nu17213335)
Supplement: Supplementary file 1 [file nutrients-17-03335-s001.zip › Supplementary file(s)/Supplementary File.pdf]

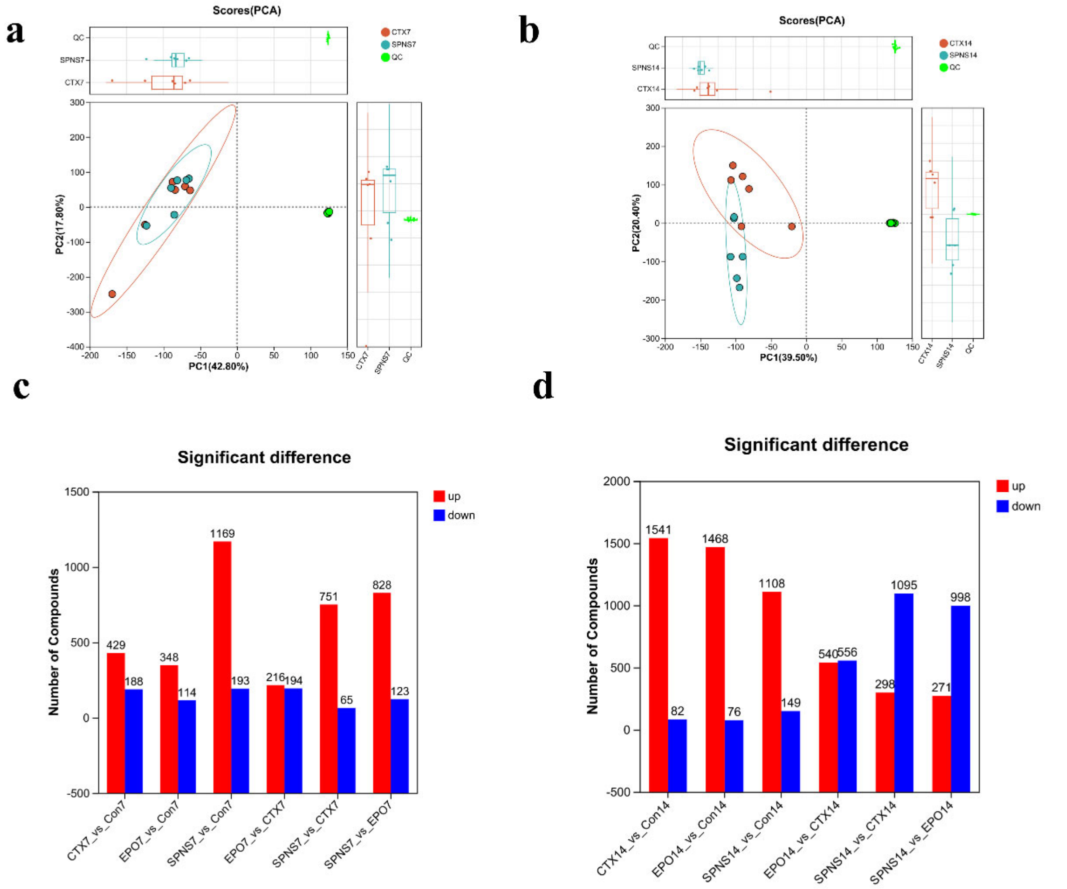

Figure S1. Metabolomic analysis of hepatic tissues. (a-b) PCA of the samples at days 7 and 14. (c-d) Histogram of differentially abundant metabolites among the groups of samples.

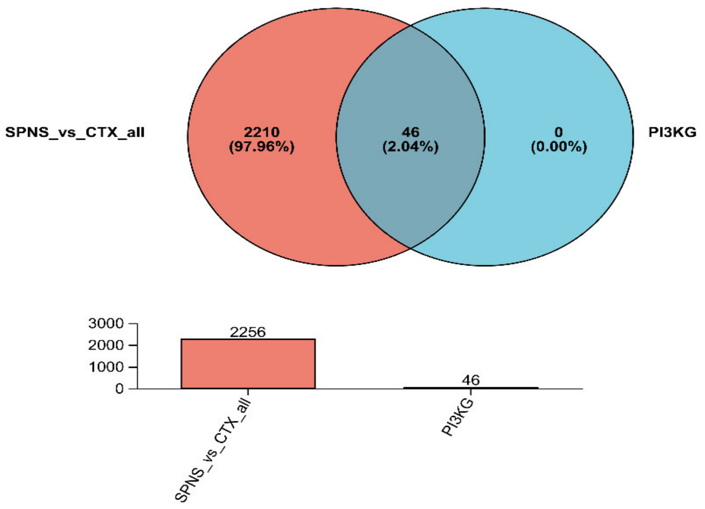

Figure S2 Proteomic analysis of hepatic tissue. Venn diagram of the differential protein sets between the SPNS and CTX groups and the protein sets of the PI3K signaling pathway.

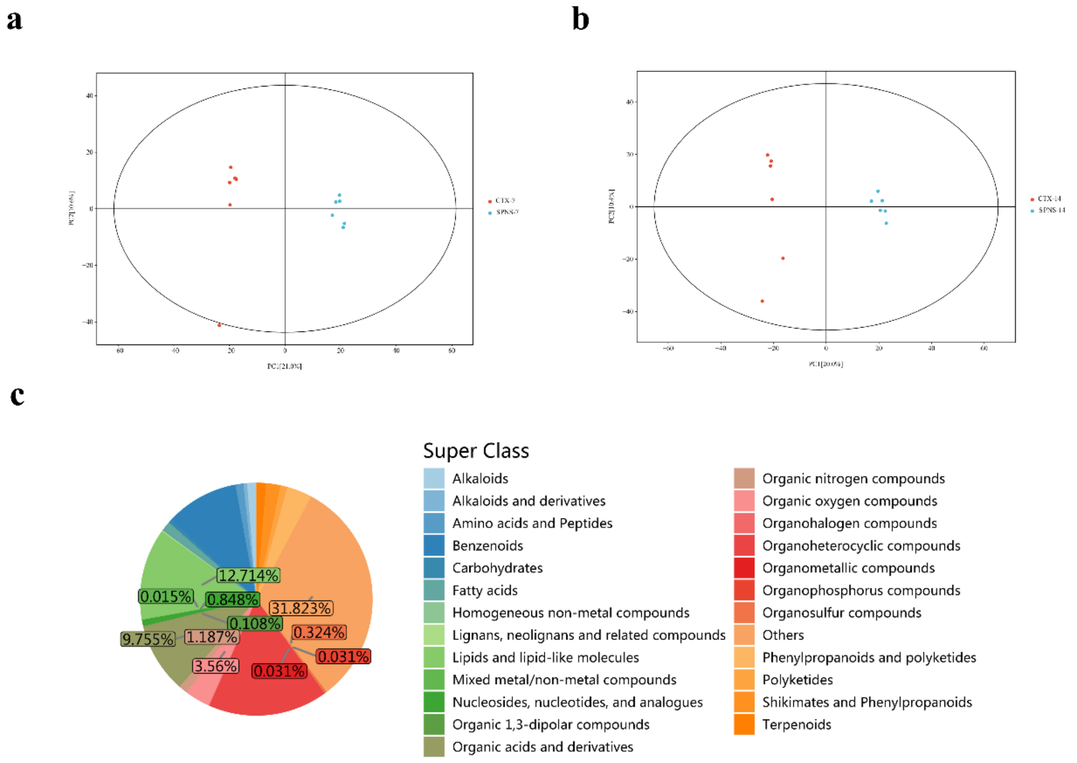

Figure S3 Metabolomic analysis of intestinal contents. (a-b) PCA of samples. (c) Metabolite classification pie chart.

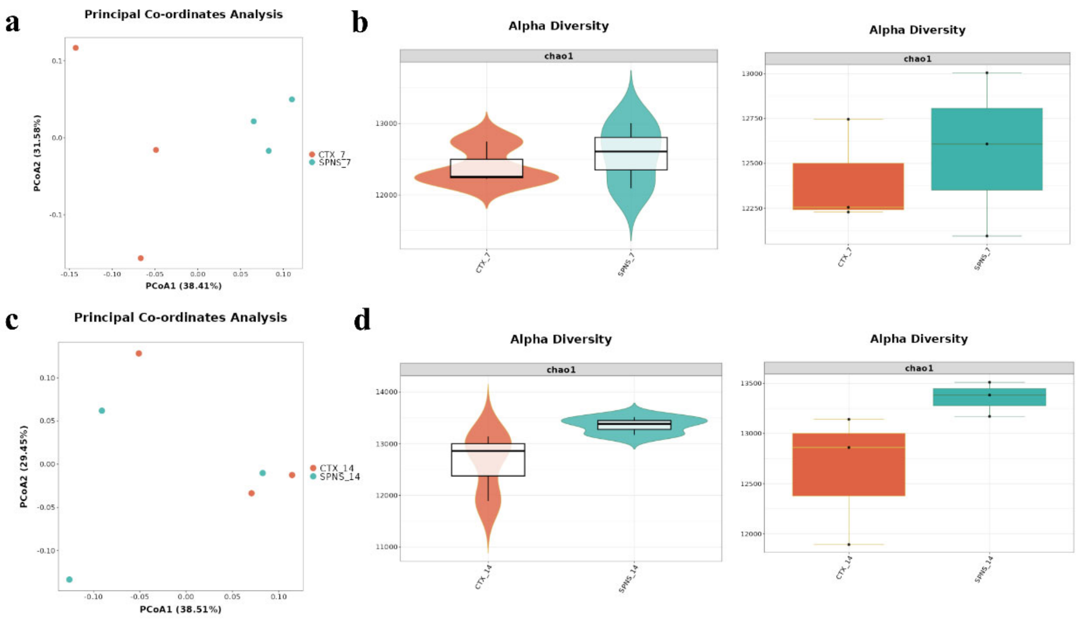

Figure S4 Gut bacterial characteristics of the CTX and SPNS groups at days 7 and 14, respectively. (a, c) PCA of samples. (b, d) Comparison of gut bacterial α diversity between the CTX and SPNS groups (Wilcoxon rank sum test).
