# Supplementary figures and images for: Steamed Panax notoginseng Saponins Ameliorate Cyclophosphamide-Induced Anemia by Attenuating Gut-Liver Injury and Activating the cAMP/PI3K/AKT Signaling Pathway
Source: Nutrients. 2025 Oct 23;17(21):3335. doi: 10.3390/nu17213335 (PMC12608177; doi:10.3390/nu17213335)

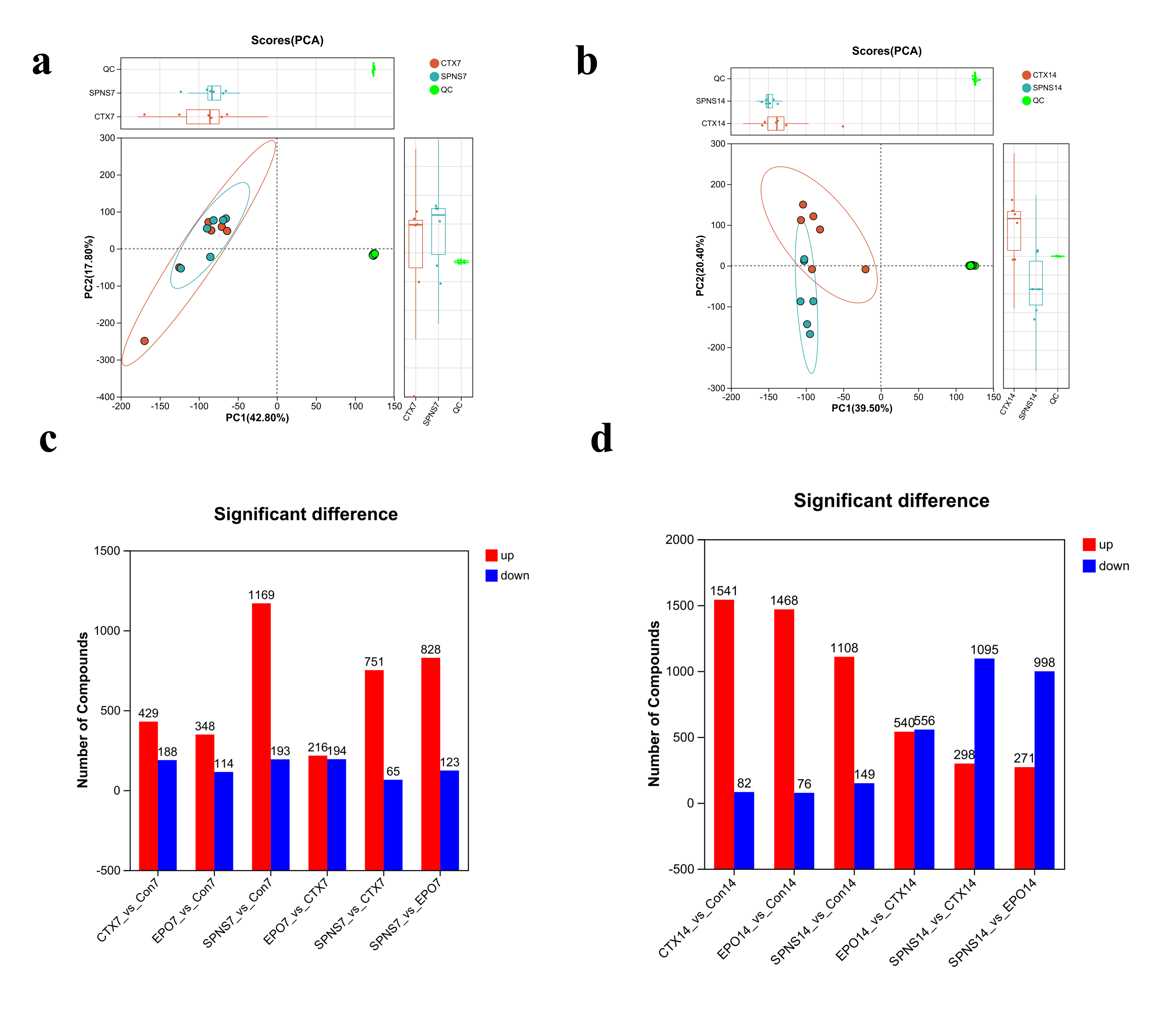

Supplement: Supplementary file 1 [file nutrients-17-03335-s001.zip › Supplementary file(s)/Figure.S1.tif]

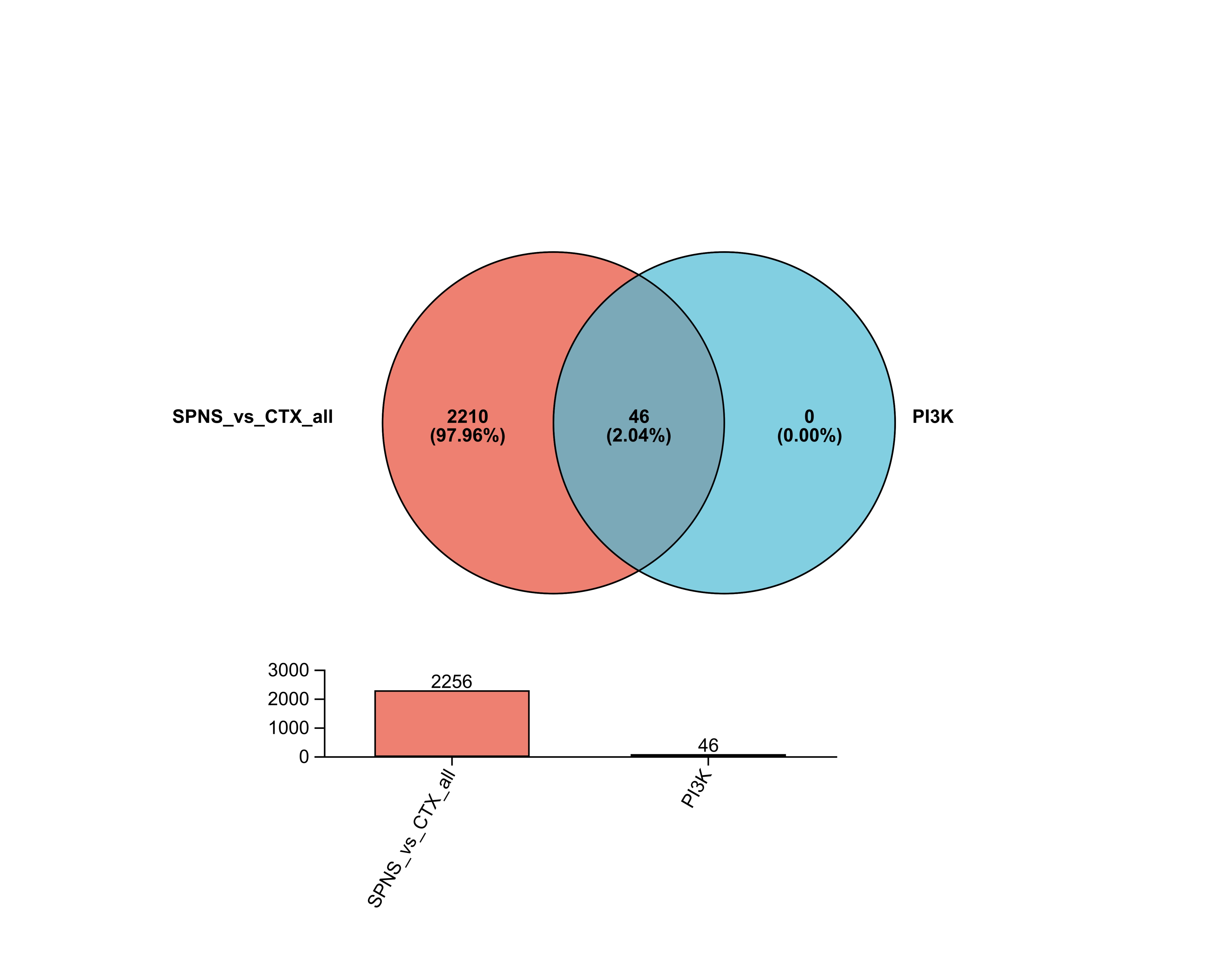

Supplement: Supplementary file 1 [file nutrients-17-03335-s001.zip › Supplementary file(s)/Figure.S2.tif]

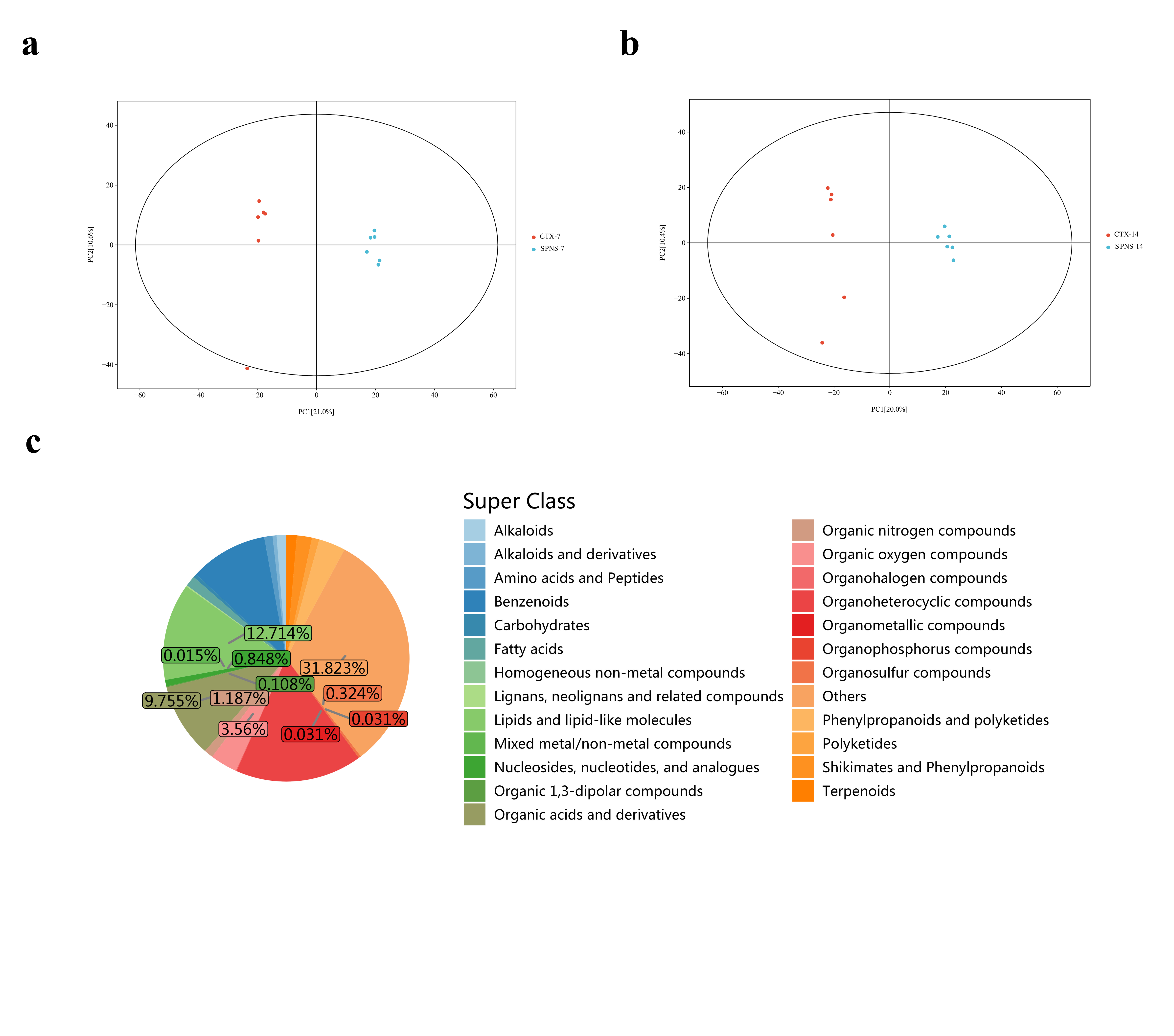

Supplement: Supplementary file 1 [file nutrients-17-03335-s001.zip › Supplementary file(s)/Figure.S3.tif]

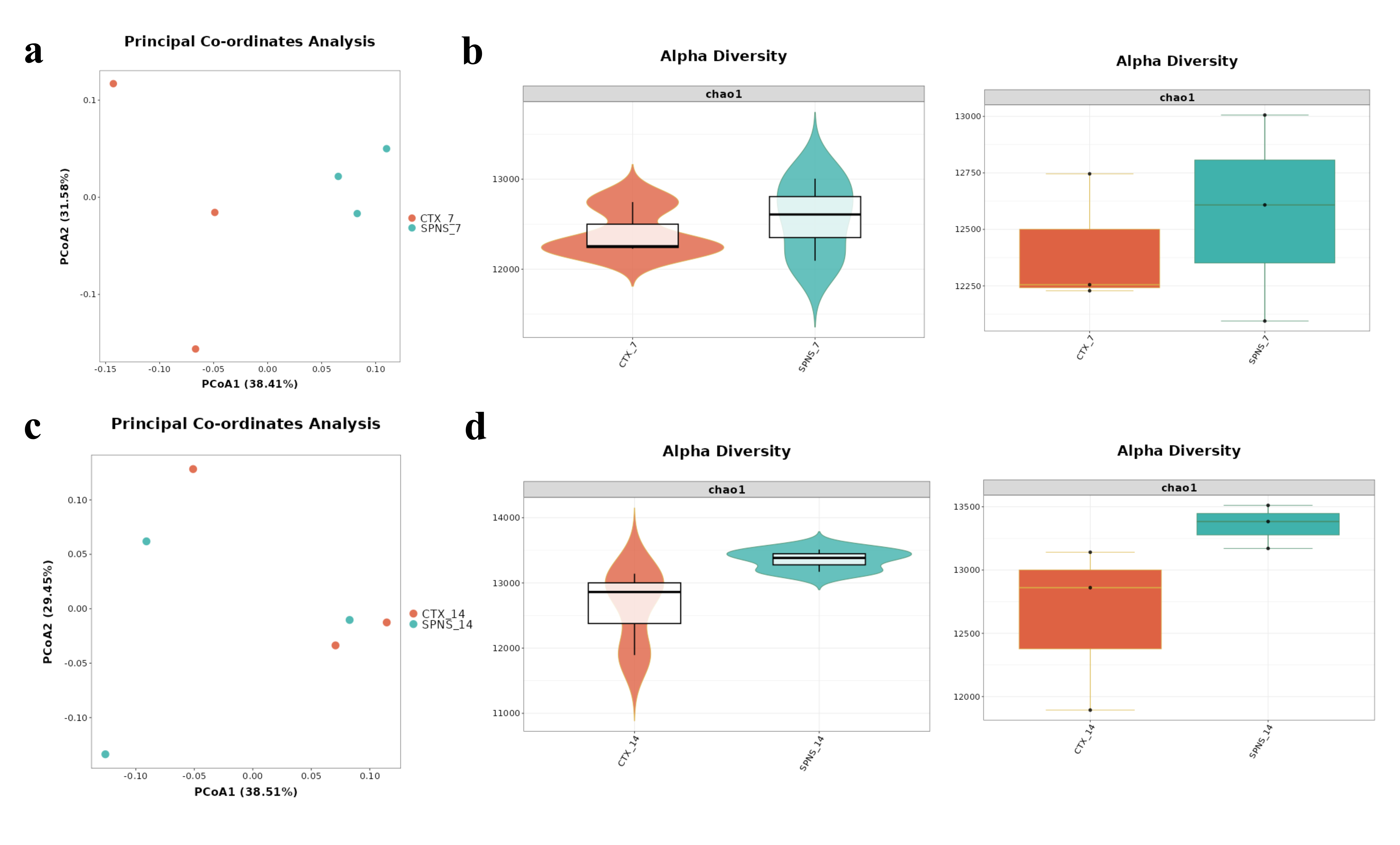

Supplement: Supplementary file 1 [file nutrients-17-03335-s001.zip › Supplementary file(s)/Figure.S4.tif]
